# Evaluating the Role of Zinc in Beta Thalassemia Major: A Prospective Case-Control Study from a Tertiary Care Teaching Hospital in India

**DOI:** 10.7759/cureus.1495

**Published:** 2017-07-20

**Authors:** Sujana Nidumuru, Venugopal Boddula, Sabitha Vadakedath, Bhagavan Reddy Kolanu, Venkataramana Kandi

**Affiliations:** 1 Biochemistry, Prathima Institute of Medical Sciences; 2 Biochemistry, Chalmeda Anand Rao Institute of Medical Sciences; 3 Department of Microbiology, Prathima Institute of Medical Sciences

**Keywords:** children, hypozincemia, beta thalassemia major, thalassemia major, zinc

## Abstract

Background

Thalassemia is a common hereditary anemia in humans, and beta thalassemia represents a group of recessively inherited hemoglobin disorders first described by Cooley and Lee and characterized by the abnormal synthesis of β-globin chain. The homozygous state results in severe anemia, which needs regular blood transfusion. Although such treatments increase the patient's life span, a variety of complications, including endocrine, metabolic, skeletal, and growth disorders are being observed due to increased iron storage in the body.

Objective

There are some reports emphasizing the role of zinc deficiency and its associated outcomes among thalassemia patients, but none from this part of the world. The aim of this study was to determine the serum zinc levels in children with beta thalassemia major.

Methods

This is a prospective case-control study, which included 35 children between the ages five and 15 years, who were diagnosed as suffering from beta thalassemia major. An equal number of age matched healthy subjects were recruited as controls. The study was carried out at the thalassemia center attached to the Prathima Institute of Medical Sciences (PIMS), Karimnagar, Telangana, India, during the year 2016. Blood samples were collected from both the cases and control subjects and serum zinc activities were analyzed using a semi-automated analyzer. Statistical Package for the Social Sciences (SPSS, Version 15.0) (SPSS Inc., Chicago, USA) was used to calculate the unpaired and independent Student's t-test (p value) to find the significance of the results.

Results

The mean concentrations of serum zinc among the cases and the controls were 39.25 ± 13.45 and 85.31 ± 13.53 (p <0.0001), respectively. Among the cases, 26 (65%) thalassemia patients had zinc concentration below 60 μg/dl, confirming hypozincemia.

Conclusion

This study revealed that hypozincemia was prevalent in beta thalassemia major patients. Further evaluation regarding the role of zinc in the development and progression of thalassemia is recommended.

## Introduction

Zinc is an essential trace element (daily requirement less than 100 mg) involved in cell proliferation, metabolism, growth, and protein synthesis [1]. There are about 300 enzymes that require zinc for their activity, but zinc majorly influences the enzyme carbonic anhydrase (CA). The activity of CA helps in knowing the zinc status of an individual, and CA is also involved in the transport of gases in the blood. Zinc exerts its function with the help of a carrier protein called metallothionein (a cysteine-rich, low molecular weight protein located in the membranes of Golgi apparatus), which also helps in the absorption and transport of zinc and copper across the duodenum. Hence, these two trace elements are interrelated. Although zinc is a trace element, it plays an important role in gene expression by forming zinc finger protein (ZFP) with four amino acids. ZFP can make small proteins interact with deoxyribonucleic acid (DNA) molecules by forming a loop with proteins and also make them bind to DNA. Thus, zinc helps in DNA-protein and protein-protein interactions, and these interactions with DNA in turn help in gene expression via regulating transcriptional factors. Zinc plays a key role in heme synthesis by activating delta-aminolevulinic acid dehydratase (ALA dehydratase). This is an enzyme that catalyzes delta-aminolevulinic acid into porphobilinogen, a precursor of heme, cytochromes, and other hemoproteins [2-3]. Zinc is one of the essential micronutrients in humans and acts as a co-factor for more than 300 enzymes. It also plays an important role in human growth and development [4]. Zinc deficiency is prevalent among children and general population belonging to developing countries as evidenced from previous reports, which also noted that dietary adjustments and supplementation could be beneficial [5-8].

Thalassemias are a group of inherited blood-borne disorders due to abnormal hemoglobin chain synthesis (either alpha or beta chain). The lack of beta chain synthesis causes beta thalassemia, which causes the body not to form new blood leading to anaemia. The abnormal hemoglobin chain synthesis requires about 200 point mutations in that particular chain of hemoglobin. Beta thalassemia major is a severe form of this blood disorder, where transfusion is the only hope for survival among patients, and a bone marrow transplant is a possible way for cure [9].

The present study is aimed at evaluating serum zinc activities among patients suffering from beta thalassemia major.

## Materials and methods

This is a prospective case-control study that included 35 children diagnosed as suffering from beta thalassemia major, attending the thalassemia unit of the Prathima Institute of Medical Sciences, Karimnagar, India. All the study subjects were between the age of five and 15 years. An equal number of age- and sex-matched control subjects, who were otherwise healthy, were also included in the study. 

The inclusion criteria were age between five and 15 years, document­ed beta thalassemia major (with hemoglobin electrophore­sis), transfusion dependency, and normal kidney and liver function tests. All the individuals with liver disease, kidney disease, gastrointestinal disease, specific dietary habits such as vegetable regimen, and consumption of zinc and minerals were excluded from the study.

After 12 hours of fasting, blood samples were collected and allowed to clot. They were later centrifuged at 3000 RPM (rotations per minute) and serum was separated. Samples were stored at -20°C, and zinc levels were estimated using commercially available kits (Coral Clinical System, Tulip Diagnostics Ltd, India) in a semiautomated clinical chemistry analyzer (Stat Fax 3000, CPC Diagnostics Pvt. Ltd., Chennai, India).

## Results

The results were drawn by analyzing the data using Microsoft Excel, and tables were generated. The measure of central tendencies (mean), and the variability (standard deviation (SD)) were calculated. The results were presented as mean ± SD, and number (%). Statistical Package for the Social Sciences (SPSS, Version 15.0) (SPSS Inc., Chicago, USA) was used to calculate the unpaired and independent Student's *t*-test (*p* value) to find the significance of the results.

The mean activities of serum zinc among beta thalassemia major cases (39.25 ± 13.45) was found to be significantly lower than the control subjects (85.31 ± 13.53), as shown in Table 1.

**Table 1:** Comparison of serum zinc activities among cases and controls *Statistically significant.

Parameter	Controls (mean ± standard deviation) n=35	Thalassemia patients (mean ± standard deviation) n=35	p Value
Serum zinc (µg/dl)	85.31 ± 13.53	39.25 ± 13.45	<0.0001*

Twenty-six (65%) of the study group children had low serum zinc activities (<60 µg/dl) highlighting severe hypozincemia in the study population.

## Discussion

Zinc is one of the essential trace elements, which has been well recognized for its role in human health [10-15]. It is important to assess the status of zinc among the general population, preferably in children [16-17]. Association of zinc and beta thalassemia major has not been adequately understood, although there are a few studies that revealed hypozincemia in beta thalassemia patients.

The present study showed decreased activities of zinc in children with beta thalassemia major. The reason for decreased zinc levels could be attributed to low dietary intake, chelation therapy, or defective absorption in the duodenum, which needs confirmation by further research.

The present study appears to be the first of its kind from this region of the world. There was one previous study from India by Bartakke, et al., who included 87 children suffering from thalassemia major. This study has noted that there was increased excretion of zinc in the urine under the effect of chelation therapy with desferrioxamine (deferoxamine) to avoid iron overload arising from repeated blood transfusions [18].

A study by Hess, et al., from Iran, which included 40 children suffering from beta thalassemia major, has noted that hypozincemia (67.35 ± 20.38 μg/dl) was prevalent and more than 65% of thalassemia patients had zinc concentrations under 70 µg/dl [6]. Another report from Iran by Mashhadi, et al., who included 333 thalassemia major patients, had observed that there was severe hypozincemia (18.36 ± 10.33 µg/dl) [19].

The results of the current study were in tune with a previous study from the United States of America by Fung, et al., who noted that there was hypozincemia (≤70 μg/dl) and also observed that zinc supplementation could improve total body bone mass among thalassemia major patients [8]. A report from Pakistan by Sultan, et al. has noted that there was a 22% prevalence of zinc deficiency (<50 μg/dl) among beta thalassemia major patients [20].

In contrast to the observations of our study and most others, a report from Pakistan by El Missiry, et al. observed that there was no disease-related zinc deficiency as evidenced from serum zinc activities of beta thalassemia major patients and their siblings [21].

In this study, we have attempted to hypothesize the potential mechanism by which zinc could directly and indirectly influence the disease course among beta thalassemia major patients.

Probable role of zinc in the development and progression of thalassemia 

In the cell, zinc-containing proteins play an important role not only in metabolic pathways but also in the regulation of gene expression by forming zinc finger protein (ZFP). ZFP helps not only in the stability of small proteins but also in the interaction with DNA. It also helps in the synthesis of proteins involved in signal transduction and cell proliferation. Dietary zinc can influence highly proliferative immune system, i.e. T-cell proliferation [22-25]. In the experimental rat models it was found that dietary zinc deficiency could decrease T-cell proliferation by reducing the availability of p56lck protein. This p56lck protein is an Src family (a family of non-receptor tyrosine kinases) protein seen on the membranes of CD4 or CD8 cells [26-28]. The other studies evaluated the cell proliferative marker enzyme thymidylate kinase (TK) in rats and found that this enzyme activity was decreased in cases of hypozincemia. TK is a pyrimidine salvage enzyme which helps in the conversion of deoxy thymidylate to deoxy thymidylate mono phosphate (dTMP), which is required for DNA synthesis. Zinc has been known to indirectly influence TK by regulating transcription; subsequently, hypozincemia was also associated with interfering with cell division by inhibiting the transformation of G1 phase (Growth 1/Gap 1 phase) of the cell cycle to S phase (Synthesis phase) [29-30].

**Figure 1:**
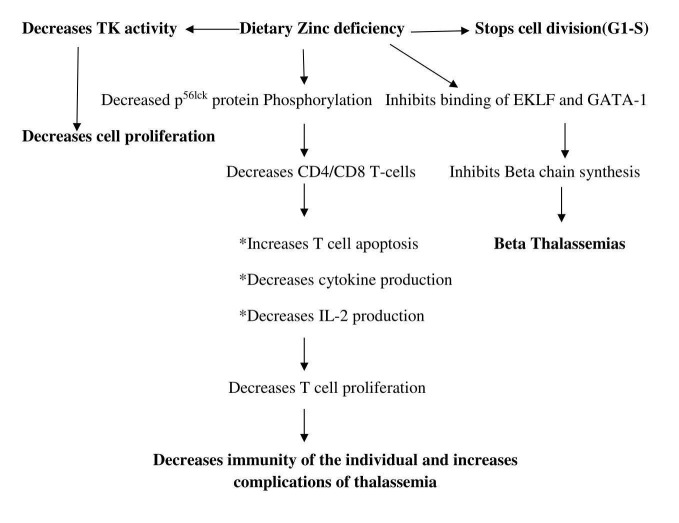
Flow chart depicting the possible mechanism of zinc in the development and progression of thalassemia CD4/ CD8: Cluster of differentiation: subsets of T lymphocytes. EKLF- erythroid Kruppel-like factor. GATA1- erythroid transcription factor/protein. IL-2- Interleukin -2. T Cell- T lymphocytes. TK- Thymidylate kinase.

The decreased zinc status of the children could be responsible for the crucial binding of erythroid Kruppel-like factor (EKLF) to the CACCC motif (box sequence of transcription) of beta chain resulting in its alteration. The defective formation of ZFP also may inhibit binding of erythroid transcriptional factor GATA-1 (erythroid transcription factor/protein) to the promoter region of the beta chain. This may result in abnormal binding of transcriptional factors to the beta gene preventing its expression or may also lead to an alteration in the base sequence of the gene (mutation). A flow chart depicting the possible mechanism of zinc in the development and progression of thalassemia is shown in Figure 1.

## Conclusions

Most cases of thalassemia are noted among paediatric age groups, causing increased morbidity. Also, repeated blood transfusions could pose a threat of transfusion-associated infectious diseases. Although genetic predisposition is key to the development of thalassemia, zinc activities and its influence on the development and progression of beta thalassemia major needs to be adequately understood. The current research results clearly demonstrate the fact that there is indeed hypozincemia among beta thalassemia major patients. Because zinc is an intrinsic part of several enzymes and because it plays a key role in cell differentiation, cell proliferation, and normal functioning of metabolism, there is an urgent need to evaluate its activities among the general population as well as in susceptible groups including thalassemia patients. Further research is required to assess the utility of zinc supplementation therapy in thalassemia patients as well as the probable role of zinc in the prevention of thalassemia.

## References

[1] Quirolo K, vichinnky E (2017). Sickle-cell disease and other haemoglobin disorders. Nelson Textbook of Pediatrics.

[2] Marengo-Rowe AJ (2007). The thalassemias and related disorders. Proc (Bayl Univ Med Cent).

[3] Galanello R, Origa R (2010). Beta-thalassemia. Orphanet J Rare Dis.

[4] Mahyar A, Ayazi P, Pahlevan A-A (2010). Zinc and copper status in children with beta-thalassemia major. Iran J Pediatr.

[5] Akhtar S (2013). Zinc status in South Asian populations—an update. J Health Popul Nutr.

[6] Hess SY, Peerson JM, King JC (2007). Use of serum zinc concentration as an indicator of population zinc status. Food Nutr Bull.

[7] Trumbo P, Yates AA, Schlicker S (2001). Dietary reference intakes: vitamin A, vitamin K, arsenic, boron, chromium, copper, iodine, iron, manganese, molybdenum, nickel, silicon, vanadium, and zinc. J Am Diet Assoc.

[8] Fung EB, Kwiatkowski JL, Huang JN (2013). Zinc supplementation improves bone density in patients with thalassemia: a double-blind, randomized, placebo-controlled trial. Am J Clin Nutr.

[9] Thein SL (2017). Molecular basis of β thalassemia and potential therapeutic targets. Blood Cells Mol Dis.

[10] Gammoh NZ, Rink L (2017). Zinc in infection and inflammation. Nutrients.

[11] Jarosz M, Olbert M, Wyszogrodzka G (2017). Antioxidant and anti-inflammatory effects of zinc. Zinc-dependent NF-κB signaling. Inflammopharmacology.

[12] Terrin G, Canani BR, Di Chiara M (2015). Zinc in early life: a key element in the fetus and preterm neonate. Nutrients.

[13] Prasad AS (2013). Discovery of human zinc deficiency: its impact on human health and disease. Adv Nutr.

[14] Maret W (2013). Zinc biochemistry: from a single zinc enzyme to a key element of life. Adv Nutr.

[15] Plum LM, Rink L, Haase H (2010). The essential toxin: impact of zinc on human health. Int J Environ Res Public Health.

[16] Cantoral A, Téllez-Rojo M, Shamah-Levy T (2015). Prediction of serum zinc levels in Mexican children at 2 years of age using a food frequency questionnaire and different zinc bioavailability criteria. Food Nutr Bull.

[17] Galetti V, Mitchikpè CE, Kujinga P (2016). Rural Beninese children are at risk of zinc deficiency according to stunting prevalence and plasma zinc concentration but not dietary zinc intakes. J Nutr.

[18] Bartakke S, Bavdekar SB, Kondurkar P (2005). Effect of deferiprone on urinary zinc excretion in multiply transfused children with thalassemia major. Indian Pediatr.

[19] Mashhadi MA, Sepehri Z, Heidari Z (2014). The prevalence of zinc deficiency in patients with thalassemia in South East of Iran, Sistan and Baluchistan province. Iran Red Crescent Med J.

[20] Sultan S, Irfan SM, Kakar J (2015). Effect of iron chelator desferrioxamine on serum zinc levels in patients with beta thalassemia major. Malaysian J Pathol.

[21] El Missiry M, Hamed Hussein M, Khalid S (2014). Assessment of serum zinc levels of patients with thalassemia compared to their siblings. Anemia.

[22] Blewett HJ, Taylor CG (2012). Dietary zinc deficiency in rodents: effects on T cell development, maturation and phenotypes. Nutrients.

[23] Pernelle JJ, Creuzet C, Loeb J (1991). Phosphorylation of the lymphoid cell kinase p56lck is stimulated by micromolar concentration of zinc. FEBS Lett.

[24] Klug A, Schwabe JW (1995). Protein motifs 5. Zinc fingers. FASEB J.

[25] Taylor CG, Giesbrecht JA (2000). Dietary zinc deficiency and expression of T lymphocyte signal transduction proteins. Can J Physiol Pharmacol.

[26] Weil R, Veillette A (1996). Signal transduction by the lymphocyte-specific tyrosine protein kinase p56lck. Curr Top Microbiol Immunol.

[27] Molina TJ, Kishihara K, Siderovski DP (1992). Profound block in thymocyte development in mice lacking p56lck. Nature.

[28] Chesters JK, Petrie L, Travis AJ (1990). A requirement for Zn2+ for the induction of thymidine kinase but not ornithine decarboxylase in 3T3 cells stimulated from quiescence. Biochem J.

[29] Prasad AS, Beck FW, Endre L (1996). Zinc deficiency affects cell cycle and deoxythymidine kinase gene expression in HUT-78 cells. J Lab Clin Med.

[30] Weatherall D (2004). The thalassemias: the role of molecular genetics in an evolving global health problem. Am J Hum Genet.

